# Association Between 2D- and 3D-Speckle-Tracking Longitudinal Strain and Cardiovascular Magnetic Resonance Evidence of Diffuse Myocardial Fibrosis in Heart Transplant Recipients

**DOI:** 10.3389/fcvm.2021.727745

**Published:** 2021-11-30

**Authors:** Wei Sun, Xuehua Shen, Jing Wang, Shuangshuang Zhu, Yanting Zhang, Chun Wu, Yuji Xie, Yun Yang, Nianguo Dong, Guohua Wang, Yuman Li, Qing Lv, Bo Liang, Li Zhang, Mingxing Xie

**Affiliations:** ^1^Department of Ultrasound, Union Hospital, Tongji Medical College, Huazhong University of Science and Technology, Wuhan, China; ^2^Hubei Province Clinical Research Center for Medical Imaging, Wuhan, China; ^3^Hubei Province Key Laboratory of Molecular Imaging, Wuhan, China; ^4^Department of Radiology, Union Hospital, Tongji Medical College, Huazhong University of Science and Technology, Wuhan, China; ^5^Department of Radiology, The Affiliated Hospital of Guizhou Medical University, Guiyang, China; ^6^Department of Cardiovascular Surgery, Union Hospital, Tongji Medical College, Huazhong University of Science and Technology, Wuhan, China

**Keywords:** heart transplant, diffuse myocardial fibrosis, cardiovascular magnetic resonance, extracellular volume fraction, speckle tracking echocardiography

## Abstract

**Objective:** This study aimed to: (1) evaluate the association between myocardial fibrosis (MF) quantified by extracellular volume fraction (ECV) and myocardial strain measured by two-dimensional (2D)- and three-dimensional speckle-tracking echocardiography (3D-STE) and (2) further investigate which strain parameter measured by 2D- and 3D-STE is the more robust predictor of MF in heart transplant (HT) recipients.

**Methods:** A total of 40 patients with HT and 20 healthy controls were prospectively enrolled. Left ventricular (LV)-global longitudinal strain (GLS), global circumferential strain (GCS), and global radial strain (GRS) were measured by 2D- and 3D-STE. LV diffuse MF was defined by cardiovascular magnetic resonance (CMR)-ECV.

**Results:** The HT recipients had a significantly higher native T1 and ECV than healthy controls (1043.8 ± 34.0 vs. 999.7 ± 19.7 ms, *p* < 0.001; 26.6 ± 2.7 vs. 24.3 ± 1.8%, *p* = 0.02). The 3D- and 2D-STE-LVGLS and LVGCS were lower (*p* < 0.005) in the HT recipients than in healthy controls. ECV showed a moderate correlation with 2D-LVGLS (*r* = 0.53, *p* = 0.002) and 3D-LVGLS (*r* = 0.60, *p* < 0.001), but it was not correlated with 2D or 3D-LVGCS, or LVGRS. Furthermore, 3D-LVGLS and 2D-LVGLS had a similar correlation with CMR-ECV (*r* = 0.60 vs. 0.53, *p* = 0.670). A separate stepwise multivariate linear analysis showed that both the 2D-LVGLS (β = 0.39, *p* = 0.019) and 3D-LVGLS (β = 0.54, *p* < 0.001) were independently associated with CMR-ECV.

**Conclusion:** CMR marker of diffuse MF was present in asymptomatic patients with HT and appeared to be associated with decreased myocardial strain by echocardiography. Both the 2D- and 3D-LVGLS were independently correlated with diffuse LVMF, which may provide an alternative non-invasive tool for monitoring the development of adverse fibrotic remodeling during the follow-up of HT recipients.

## Introduction

Heart transplant (HT) is the most effective treatment for patients with end-stage heart failure (1). The outcome of HT has significantly improved owing largely to advances in immunosuppressive therapy and the management of long-term complications. However, myocardial fibrosis (MF) is a commonly demonstrated histopathologic feature and is associated with a higher risk of adverse cardiac events and death after HT (2–9). Therefore, a reliable non-invasive method for monitoring the development of fibrotic remodeling early may be desirable during the follow-up of patients with HT and can assist to risk stratification in HT recipients.

Histological biopsy is the gold standard for the assessment of MF, but it is invasive and not practical for serial follow-up after HT. Besides, the biopsied region only reflects the local pathological information and cannot quantify diffuse MF. However, numerous studies have validated that diffuse MF can be measured non-invasively by extracellular volume fraction (ECV) that was derived from cardiovascular magnetic resonance (CMR) in various cardiovascular diseases (10–13). Furthermore, previous studies have reported that two-dimensional speckle-tracking echocardiography (2D-STE)-derived myocardial strain may be a robust indicator to predict MF (14–16). However, 2D-STE is time-consuming and is affected by the through-plane motion during a cardiac cycle and the transplanted hearts would show noteworthy translational motion during the cardiac phase, which can aggravate the “out-of-plane phenomenon” of 2D-STE. Three-dimensional (3D)-STE can overcome the limitation of plane dependency existing in 2D-STE. Moreover, 3D-STE requires less time to acquire and analyze images (17). Nevertheless, the predictive value of myocardial strain for diffuse MF in HT recipients and whether 3D-STE is superior to 2D-STE remained unknown.

Therefore, this study aimed to: (1) evaluate the association between MF and myocardial strain measured by 2D- and 3D-STE and (2) further investigate which strain parameter measured by 2D- and 3D-STE is the more robust predictor of MF defined by CMR-ECV in HT recipients.

## Materials and Methods

### Study Population

A total of 50 asymptomatic patients with HT who were scheduled for echocardiogram at their routine follow-up examinations were prospectively enrolled in this study. As myocardial edema due to inflammation can expand the extracellular volume and increase native T1 times, patients with acute rejection (AR) at the time of recruitment were excluded. Patients with the following conditions were also excluded: time after HT <6 months, left ventricular ejection fraction (LVEF) <50%, significant coronary allograft vasculopathy (CAV), uncontrolled hypertension, uncontrolled blood glucose, renal failure, arrhythmia, and poor image quality. There were 10 patients excluded due to poor echocardiographic image quality (*n* = 6) and arrhythmia (*n* = 4). The remaining 40 patients were included in the final analysis. Demographic data including sex, age, height, and weight at the time of echocardiographic examination were obtained. Clinical data including recipient age, weight at HT, bypass time, ischemia time, donor age, weight, and history of AR and CAV were collected from a medical record review. Additionally, 20 healthy volunteers with a similar distribution of sex and age to the HT group were recruited as the control group. They had no history of hypertension, diabetes mellitus, renal failure, or other organic diseases based on physical examinations, biochemical tests, ECG, and echocardiography that were enrolled as a control group.

This study was approved by the Ethics Committee of Tongji Medical College, Huazhong University of Science and Technology, and all the participants provided a written informed consent.

### Conventional 2D Echocardiography

All the 2D, Doppler, and 3D images were acquired by using a commercially available system (EPIQ 7C, Philips Medical Systems, Andover, Massachusetts, USA) with S5-1 and X5-1 transthoracic echocardiography transducers. Four heartbeat images were collected and stored in digital format. All the echocardiographic parameters were acquired according to the published guidelines (18). LV diameter was measured at end-diastole from the parasternal long-axis. Doppler mitral valve peak early (E) and late (A) diastolic velocities and E/A velocity ratio were measured on apical four-chamber view. The mean value of early diastolic mitral annular tissue velocity and left ventricular lateral wall tissue velocity (e') was measured by using tissue Doppler imaging. The LV Tei index was defined as the ratio of isovolumic time divided by ejection time with the use of tissue Doppler of the mitral annulus.

### Left Ventricular Myocardial Strain Measured by 2D-STE and 3D-STE

**Figure 1:**
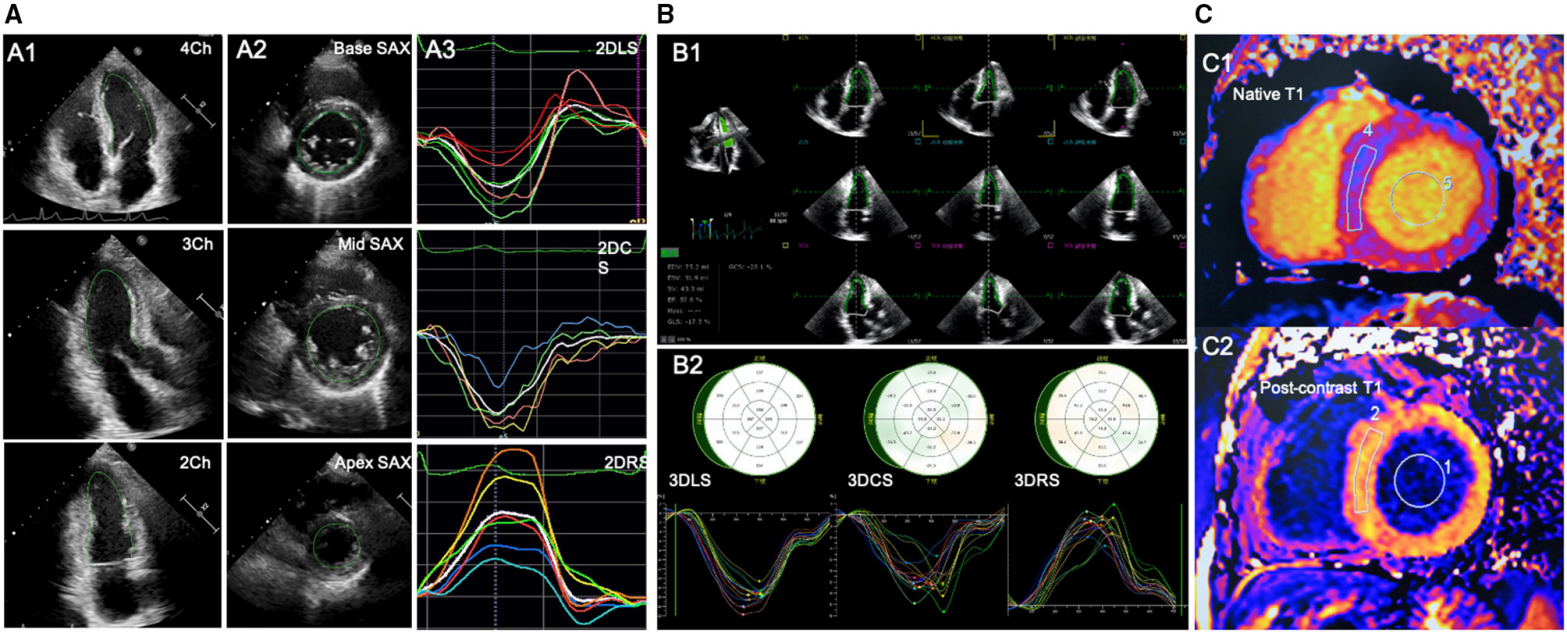
Offline analysis of STE for left ventricular (LV) strain and CMR for LV-ECV. **(A)** 2D-STE offline analysis for LV. A1-A2, LV endocardial tracing in the three LV apical long-axis and three LV short-axis views; A3, LV strain curves; **(B)** 3D-STE offline analysis for LV. B1, LV endocardial tracing at end-diastole and end-systole; B2, 3D-GLS, GCS, and GRS strain curves were generated. **(C)** Offline analysis for CMR-LV-ECV. C1-C2, measurements of native and postcontrast T1 time from LV myocardium and LV blood pool. 2D-STE, two-dimensional speckle-tracking echocardiography; 3D-STE, three-dimensional STE; CMR, cardiovascular magnetic resonance; ECV, extracellular volume fraction; GLS, global longitudinal strain; GCS, global circumferential strain; GRS, global radial strain.

The 2D myocardial strain was performed on the 2D cardiac performance analysis 1.2 software (TomTec Imaging Systems, Unterschleissheim, Germany, UK) in the 2D echocardiographic images with a frame rate of 50–70 MHz. LV longitudinal strain was determined by endocardial tracing in the apical four-, three-, and two-chamber views. LV circumferential and radial strain were measured by endocardial tracing in the basal, middle, and apical level of LV short-axis views on a frame-by-frame basis during one cardiac cycle. If the tracking was suboptimal, manual adjustment was performed. The LV global longitudinal strain (LVGLS), LV global circumferential strain (LVGCS), and LV global radial strain (LVGRS) were defined as the average peak strain values automatically generated from the 16 segmental strain curves by the software (Figure 1A).

The 3D-STE was performed on the four-dimensional (4D) LV-Analysis 3.1 Software (TomTec Imaging systems, Unterschleissheim, Germany, UK) in the 3D LV full-volume images with frame rate of 19–23 MHz. After selecting the center of the mitral annulus line and the apex of the left ventricle at end-diastole, the workstation would track the LV endocardium automatically and the manual adjustment was performed in case of unsatisfactory tracking. The same procedure was performed at the end-systolic frame. Subsequently, the software would perform the 3D-STE throughout the cardiac cycle. Ultimately, the 3D-LV-end-diastolic volume (EDV), end-systolic volume (ESV), LVEF, and the myocardial strain would be generated automatically. LVGLS, LVGCS, and LVGRS, respectively, were calculated as the average peak systolic longitudinal, circumferential, and radial strain of all the 16 LV segments (Figure 1B). Subjects with two or more inadequately tracked segments were removed from the analysis.

### Cardiovascular Magnetic Resonance Images Acquired and Analyzed

Cardiovascular magnetic resonance imaging was performed with a 1.5-Tesla system (MAGNETOM Aera, Siemens Healthineers, Erlangen, Germany, UK). CMR examination was performed within 24 h of the echocardiography examination. Three long axes and a set of contiguous short-axis cine images of LV were acquired with a steady-state free precession sequence during breath-hold of 10–15 s. The cine image parameters were as follows: repetition time (TR)/echo time (TE), 38.09/1.21 ms; slice thickness, 8 mm; field of view, 340 × 255 mm^2^; matrix, 256 pixels × 205 pixels; and flip angle, 80°. T1 mapping was performed on three standard LV short-axis slices before and 15 min after the administration of a bolus of gadopentetate dimeglumine contrast agent (0.2 mmol/kg, Magnevist, Bayer Healthcare, Berlin, Germany, UK) by using a modified Look-Locker inversion recovery (MOLLI) sequence with a 5- and 3-sampling scheme. The typical MOLLI sequence parameters were: TR/TE, 255.76/1.01 ms; slice thickness, 8 mm; matrix, 144 × 256 pixels; flip angle, 35°; and scan time, 11 s. The parameters of postcontrast T1 were similar to those of the native T1, except for a TE of 1.12 ms and a TR of 359.76 ms. Image acquisitions at LV basal, middle, and apical short-axis slices in diastole were performed before and 15 min after the injection of gadolinium, respectively.

Cardiovascular magnetic resonance data were analyzed with a commercial software (Argus, Siemens Healthineers, Erlangen, Germany, UK) by an experienced reader blinded to the echocardiographic results. Regions of interest were drawn in the blood and a midwall region of the myocardium and copied between the pre- and postcontrast T1 and ECV maps. To measure the T1 value of blood, a circular region of interest was positioned in the LV cavity, avoiding papillary muscle (Figure 1C). Then, the ECV was calculated from the native and postcontrast T1 time, along with a hematocrit correction that was obtained on the same day of the CMR scanning according to the well-established formula (19).

### Reproducibility

To evaluate the reproducibility of 2D-STE and 3D-STE measurements, a total of 20 subjects (including 10 HT recipients and 10 healthy controls) were selected randomly. For intraobserver variability, analysis of the first 2D-STE and 3D-STE data set of the 20 subjects (including 10 HT recipients and 10 healthy controls) was repeated 2–4 weeks later by the same primary investigator. For interobserver variability, the data set was analyzed by two blinded investigators.

### Statistical Analysis

Continuous variables are presented as mean ± SD or median [interquartile range (IQR)] and categorical variables are presented as absolute numbers (percentages). The unpaired two-sample *t*-test or the Mann–Whitney *U*-test was used to compare continuous variables and the chi-squared test or the Fisher's exact test was used to compare categorical variables between the HT and control group. Correlations between continuous variables were evaluated with the Pearson's correlation coefficients. Potential predictors (demographic, clinical, LV conventional function parameters, and myocardial strains) of ECV in HT recipients were identified by the univariate linear regression analysis. The variables with univariate *p* < 0.10 were included in the multivariate linear regression analysis. To avoid collinearity issues, the multivariate stepwise linear regression analysis for 2D-LVGLS and 3D-LVGLS (model 1 for 2D-LVGLS and model 2 for 3D-LVGLS) was performed separately, along with those echocardiographic and clinical variables that demonstrated a univariate *p* < 0.10. The interobserver and intraobserver variability of the 10 HT recipients and 10 healthy volunteers were assessed by the intraclass correlation coefficients (ICCs) and the Bland–Altman analyses. Comparison of correlation coefficients was performed with the MedCalc version 19.0.4 (MedCalc Software, Ostend, Belgium, UK). All the statistical analyses, except for the comparison of correlation coefficients, were performed with SPSS Statistics version 23.0 (IBM, Armonk, New York, USA). *p* < 0.05 was considered as statistically significant.

## Results

### Clinical Characteristics

The baseline clinical characteristics of the 40 patients with HT are given in Tables 1, 2. The most prominent etiology for HT was dilated cardiomyopathy. The echocardiographic studies occurred at a median of 1.2 years after HT (IQR: 1.0–2.9 years). No patients had clinically significant AR or CAV when they were enrolled in this study. Immunosuppression regimens varied, but tacrolimus, mycophenolate mofetil, and prednisone were commonly used. Aspirin and statin were also frequently used.

**Table 1:** Clinical characteristics of the 40 patients with HT.

**Parameters**	**Value**
**Characteristics Pre-HT**	
Recipient	
Men, *n* (%)	30 (75)
Age, (years)	43 ± 13
Weight, (kg)	58 (50, 65)
Etiology for transplantation, *n* (%)	
Dilated cardiomyopathy	24 (60)
Ischemic cardiomyopathy	8 (20)
Valvular heart disease	3 (7.5)
Hypertrophic cardiomyopathy	1 (2.5)
Complex congenital heart disease	1 (2.5)
Other disease	3 (7.5)
Invasive mPAP, (mmHg)	38 ± 14
Bypass time, (min)	97 (83, 115)
Aortic clamp time (min)	30 (27, 37)
Donor	
Age, (years)	35 ± 10
Weight, (kg)	65 (55, 65)
Weight ratio, (donor/recipient)	1.1 ± 0.3
Ischemic time, (min)	333 (195, 380)
**Characteristics at echocardiographic examination**	
Time since transplantation, (years)	1.2 (1.0, 2.9)
Surgical technique	
Biatrial technique, *n* (%)	14 (35)
Bicaval technique, *n* (%)	26 (65)
History of AR, *n* (%)	4 (10)
Immunosuppressant medications	
Tacrolimus, *n* (%)	35 (87.5)
Sirolimus/everolimus, *n* (%)	6 (15.0)
Cyclosporine, *n* (%)	5 (12.5)
Mycophenolate mofetil, *n* (%)	37 (92.5)
Prednisone, *n* (%)	32 (80.0)
Aspirin, *n* (%)	26 (65.0)
Statin, *n* (%)	25 (62.5)
ACE inhibitor/ARB, *n* (%)	15 (37.5)
Calcium-channel blocker, *n* (%)	18 (45.0)
Beta-blocker, *n* (%)	18 (45.0)

*AR, acute rejection; HT, heart transplant; mPAP, mean pulmonary arterial pressure*.

**Table 2:** Demographic information, echocardiographic, and CMR findings of the HT group and control group.

**Parameters**	**Control group (*n* = 20)**	**HT group (*n* = 40)**	***P*-value**
**Demographic information at echocardiography examination**			
Men (%)	14 (70%)	34 (75%)	0.68
Age (years)	45 ± 11	45 ± 14	0.944
Height (cm)	168 ± 8	166 ± 8	0.482
Weight (kg)	65 ± 6	62 ± 13	0.071
BSA (m^2^)	1.7 ± 0.1	1.7 ± 0.2	0.118
BMI (kg/m^2^)	23 (21, 24)	22 (20, 24)	0.169
Obesity, *n* (%)	3 (15.0)	8(20.0)	0.637
HR (bpm)	67 ± 10	88 ± 7	<0.001
SBP (mmHg)	120 (110, 124)	120 (112, 126)	0.346
DBP (mmHg)	80 (70, 87)	80 (71, 85)	0.766
**Echocardiographic findings**			
LA, (mm)	33 ± 3	46 ± 7	<0.001
LVd, (mm)	44 ± 2	42 ± 4	0.093
IVST, (mm)	8.9 ± 0.8	9.6 ± 1.3	0.01
LVMI, (g)	72.0 ± 11.1	82.5 ± 17.6	0.007
Mitral valve			
E, (m/s)	0.7 ± 0.2	0.8 ± 0.2	0.366
A, (m/s)	0.6 ± 0.1	0.5 ± 0.1	<0.001
E/A ratio	1.2 ± 0.4	1.8 ± 0.4	<0.001
e', (cm/s)	11 ± 2	11 ± 2	0.528
E/e'	7 ± 2	8 ± 3	0.334
DT, (ms)	210 ± 45	182 ± 34	0.02
Tei index	0.36 ± 0.06	0.52 ± 0.11	<0.001
2D-LVGLS, (%)	−20.0 ± 2.5	−16.9 ± 2.3	<0.001
2D-LVGCS, (%)	−29.1 ± 3.3	−26.6 ± 4.7	0.038
2D-LVGRS, (%)	39.3 ± 6.0	37.8 ± 7.6	0.198
3D-LVEDV, (ml)	90.5 ± 17.8	72.0 ± 19.4	0.001
3D-LVESV, (ml)	30.7 ± 7.3	27.2 ± 9.3	0.013
3D-LVEF, (%)	66.1 ± 3.9	62.4 ± 5.5	0.008
3D-LVGLS, (%)	−20.8 ± 1.5	−17.2 ± 2.3	<0.001
3D-LVGCS, (%)	−31.5 ± 3.7	−28.3 ± 5.1	0.015
3D-LVGRS, (%)	42.3 ± 4.1	40.7 ± 6.3	0.339
CMR findings^*^			
Native T1 time, (ms)	999.7 ± 19.7	1043.8 ± 34.0	<0.001
Post-contrast T1 time, (ms)	455.3 ± 24.5	429.8 ± 39.7	0.013
ECV, *n* (%)	24.3 ± 1.8	26.6 ± 2.7	0.002

*2D, two dimensional; 3D, three dimensional; A, late diastolic inflow velocity; BSA, body surface area; CMR, cardiovascular magnetic resonance; DBP, diastolic blood pressure; DT, deceleration time of E; E, early diastolic inflow velocity; e', mean value of early diastolic mitral annular tissue velocity and left ventricular lateral wall tissue velocity; ECV, extracellular volume fraction; HR, heart rate; IVST, interventricular septum thickness; LA, left atrial diameter; LVd, left ventricular diastolic diameter; LVMI, left ventricular mass indexed to body surface area; LVEF, left ventricular ejection fraction; LVGLS, left ventricular global longitudinal strain; LVGCS, left ventricular global circumferential strain; LVGRS, left ventricular global radial strain; PA, pulmonary artery; SBP, systolic blood pressure*.

* *Means that 31 patients with HT and 20 healthy controls had the data of CMR findings*.

### Echocardiographic and CMR Findings

**Figure 2:**
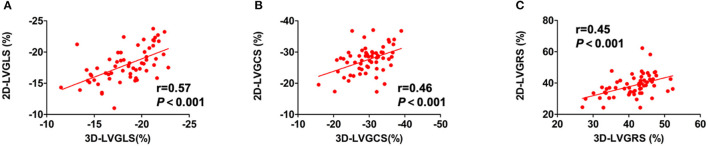
The relationships between LV strain parameters from 2D-STE and 3D-STE. The relationship between **(A)** 2D-LVGLS and 3D-LVGLS, **(B)** 2D-LVGCS and 3D-LVGCS, and **(C)** 2D-LVGRS and 3D-LVGRS. 2D-STE, two-dimensional speckle-tracking echocardiography; 3D-STE, three-dimensional STE; GLS, global longitudinal strain; GCS, global circumferential strain; GRS, global radial strain; LV, left ventricular.

Comparisons of echocardiographic and CMR parameters in the 40 patients with HT and 20 healthy controls are shown in Table 2. Compared with healthy controls, patients with HT had a significantly greater E/A ratio of the mitral valve and shorter deceleration time (DT) of E wave, which may suggest impaired LV diastolic function. Concerning LV systolic function, patients with HT had lower 3D-LVEF than the healthy controls (62.4 ± 5.5 vs. 66.1 ± 3.9%, *p* = 0.008), but remained within the normal range. However, HT group showed impaired 2D-LVGLS, 2D-LVGCS, 3D-LVGLS, and 3D-LVGCS. In contrast, the two groups did not differ in 2D- and 3D-LVGRS. Moreover, a weak to moderate but significant correlation of global strain values between the 2D- and 3D-STE was noted in the entire study population (LVGLS: *r* = 0.57, *p* < 0.001; LVGCS: *r* = 0.46, *p* < 0.001; LVGRS: *r* = 0.45, *p* < 0.001) (Figure 2). There were 31 of the 40 patients with HT and all the 20 healthy controls agreed to undergo CMR examination within 24 h of the echocardiography examination. Compared with healthy controls, the HT recipients showed significantly longer native T1 time (999.7 ± 19.7 vs. 1043.8 ± 34.0 ms, *p* < 0.001) and greater ECV (24.3 ± 1.8 vs. 26.6 ± 6.7%, *p* = 0.002).

### Relationships Between Diffuse LVMF and Clinical and Echocardiographic Parameters

**Figure 3:**
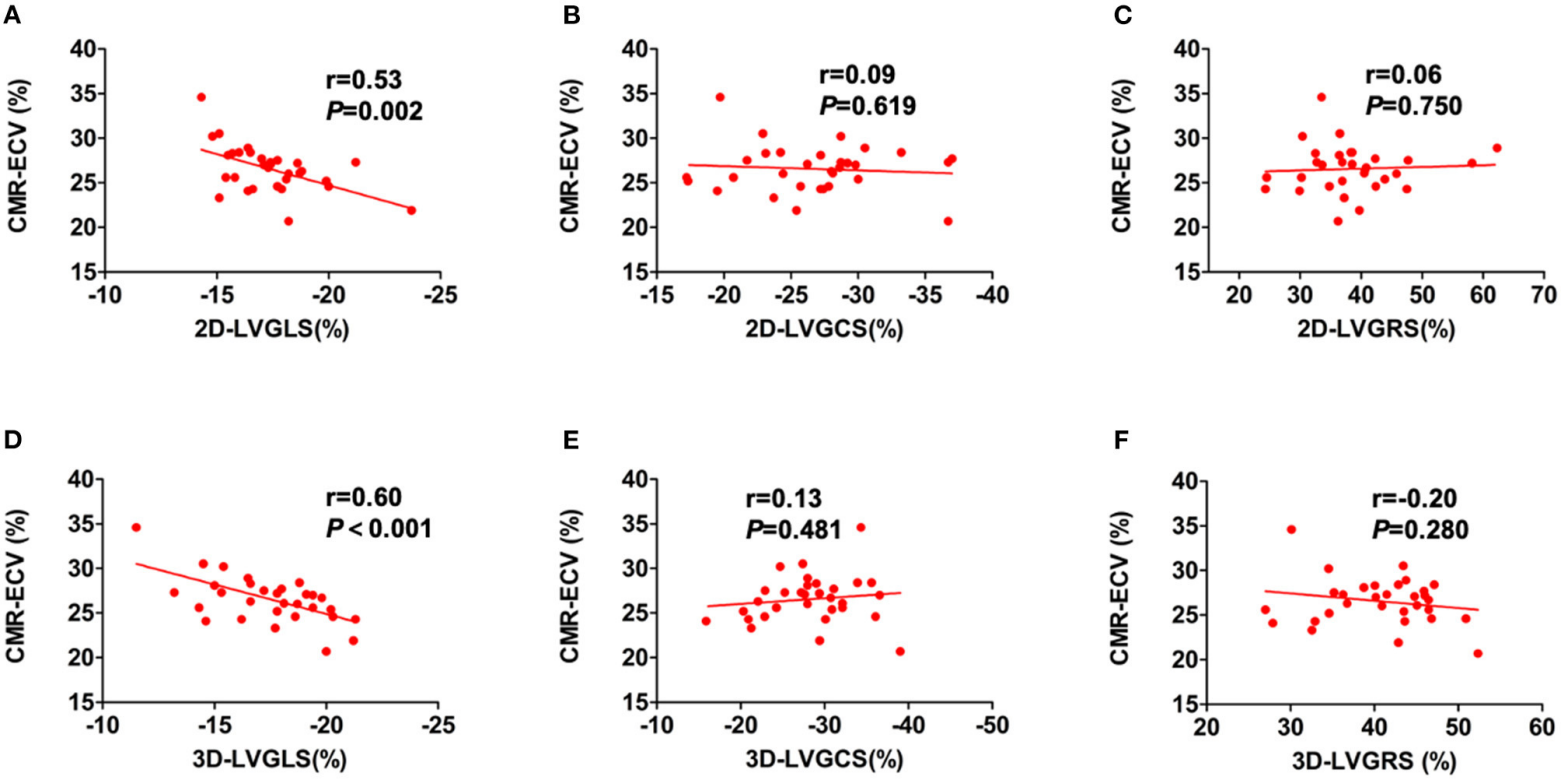
The correlations of CMR-ECV and LV strain parameters. Correlations were showed between CMR-ECV and 2D-LVGLS **(A)**, 2D-LVGCS **(B)**, 2D-LVGRSV **(C)**, 3D-LVGLS **(D)**, 3D-LVGCS **(E)**, and 3D-LVGRSV **(F)**. 2D, two-dimensional; 3D, three-dimensional; CMR, cardiovascular magnetic resonance; ECV, extracellular volume fraction; GLS, global longitudinal strain; GCS, global circumferential strain; GRS, global radial strain; LV, left ventricular.

The diffuse LVMF was defined by CMR-ECV in this study. The correlations between LV myocardial strain and CMR-ECV are shown in Figure 3. For 2D-LV myocardial strain, only 2D-LVGLS correlated with ECV (*r* = 0.53, *p* = 0.002), whereas LVGCS and LVGRS showed no correlation with ECV. Figure 3 also illustrates the association of 3D-LV myocardial strain with CMR-ECV. Similarly, as shown in Figures 3D–F, ECV only had correlation with 3D-LVGLS (*r* = 0.60, *p* < 0.001) and not had correlation with 3D-LVGCS or 3D-LVGRS. Moreover, 3D-LVEF did not correlate with CMR-ECV. Furthermore, there was no significant difference between the correlation of 2D-LVGLS with CMR-ECV and the correlation of 3D-LVGLS with CMR-ECV (*r* = 0.53 vs. r = 0.60, *p* = 0.670).

Table 3 shows the results of the univariate and multivariate linear regression analyses for the relationship between ECV and the clinical and echocardiographic variables in the HT recipients. The univariate regression analysis revealed that recipient age, time since HT, DT, 2D-LVGLS, and 3D-LVGLS were significantly correlated with ECV. In addition, 2D-LVGLS had correlation with 3D-LVGLS (*r* = 0.57, *p* < 0.001) (Figure 2). Therefore, to avoid the problems of collinearity, 2D-LVGLS and 3D-LVGLS were evaluated in two separate models. Finally, the stepwise multivariate regression analysis showed that 2D-LVGLS (β = 0.39, *p* = 0.019) and 3D-LVGLS (β = 0.54, *p* < 0.001) both were independently associated with CMR-ECV.

**Table 3:** Univariate and multivariate linear regression analysis for ECV in patients with HT (*n* = 31).

	**Univariate analysis**		**Multivariate analysis**			
			**Model 1 + 2D-LVGLS**		**Model 2 + 3D-LVGLS**	
**Variables**	**β**	***P*-value**	**β**	***P*-value**	**β**	***P*-value**
**Pre-HT**						
Donor age, (years)	0.21	0.280				
Donor weight, (kg)	−0.08	0.663				
Recipient age, (years)	0.37	0.044	0.18	0.267	0.09	0.543
Recipient weight, (kg)	0.17	0.378				
Weight ratio, (donor/recipient)	−0.02	0.923				
Invasive mPAP, (mmHg)	0.21	0.276				
CPB time, (min)	0.10	0.592				
Aortic clamp time, (min)	0.11	0.559				
Ischemic time, (min)	−0.07	0.737				
**Post-HT**						
HR, (bpm)	−0.13	0.496				
SBP, (mmHg)	−0.24	0.192				
DBP, (mmHg)	−0.22	0.233				
History of ACR, (yes vs no)	−0.19	0.302				
Time since transplantation, (years)	0.42	0.018	0.24	0.139	0.32	0.022
Mitral valve						
E/A ratio	0.04	0.822				
E/e'	0.17	0.376				
DT, (ms)	−0.32	0.079	−0.23	0.134	−0.29	0.039
LV Tei index	0.16	0.398				
2D-LVGLS, (%)	0.53	0.002	0.39	0.019		
2D-LVGCS, (%)	0.09	0.619				
2D-LVGRS, (%)	0.06	0.750				
3D-LVEF, (%)	0.26	0.151				
3D-LVGLS, (%)	0.60	<0.001			0.54	<0.001
3D-LVGCS, (%)	0.13	0.481				
3D-LVGRS, (%)	−0.20	0.280				
3D-LVEF, (%)	−0.26	0.151				

*Abbreviations as in Tables 1, 2*.

### Reproducibility

The measurements obtained by 2D-STE and 3D-STE showed excellent reproducibility. The complete data of interobserver variability and intraobserver variability were shown in Table 4.

**Table 4:** Intraobserver and interobserver reproducibility for the parameters of the two-dimensional and the three-dimensional speckle-tracking echocardiography.

	**ICC (95% CI)**	**Bias**	**Limits of agreement**
**Intra-observer (*****n*** **= 20)**
2D-LVGLS, (%)	0.95 (0.88-0.98)	0.1	−1.5~1.7
2D-LVGCS, (%)	0.94 (0.85-0.97)	0.2	−3.0~3.3
2D-LVGRS, (%)	0.87 (0.71-0.95)	1.0	−6.7~4.7
3D-LVGLS, (%)	0.97 (0.93-0.99)	0.1	−1.2~1.2
3D-LVGCS, (%)	0.92 (0.80-0.97)	0.3	−3.7~3.2
3D-LVGRS, (%)	0.90 (0.78-0.96)	0.8	−3.7~5.3
**Inter-observer (*****n*** **= 20)**
2D-LVGLS, (%)	0.93 (0.83-0.97)	0.1	−1.7~2.0
2D-LVGCS, (%)	0.90 (0.76-0.96)	1.2	−2.7~5.3
2D-LVGRS, (%)	0.85 (0.67-0.94)	1.3	−7.3~4.8
3D-LVGLS, (%)	0.95 (0.88-0.98)	0.1	−1.6~1.8
3D-LVGCS, (%)	0.87 (0.70-0.95)	0.3	−4.4~4.0
3D-LVGRS, (%)	0.86 (0.67-0.94)	0.8	−4.6~6.2

*Abbreviations as in Table 2*.

*n = 20 means that there were 10 patients with HT and 10 healthy controls data used in this table*.

## Discussion

To the best of our knowledge, this is the first study to evaluate the level of diffuse LVMF with non-invasive CMR-ECV, further investigating the predictive value of 3D-STE-derived LV strain for diffuse LVMF in patients with post-HT, and directly compares its utility with that of 2D-STE-derived strains. The main findings of this study were: (1) the level of diffuse MF defined by CMR-ECV was higher even in asymptomatic patients with HT than in healthy controls; (2) the increased LVMF measured by CMR-ECV was correlated with 2D- and 3D-LVGLS, not correlated with LV-GCS, GRS, or ejection fraction (EF); and (3) both the 2D- and 3D-LVGLS were independently correlated with the extent of diffuse LVMF in HT recipients.

### Left Ventricular MF in Patients With HT

The MF is a commonly demonstrated histopathologic feature in patients with HT and its correlation with a higher risk of adverse clinical outcome has been validated by the previous studies (2–5). Adverse fibrotic myocardial remodeling is a suspected long-term sequela of HT recipients. Therefore, it is clinically crucial to quantify the MF in the transplanted hearts at an early stage.

Histological biopsy is the gold standard for assessing MF, but it is invasive. The myocardial samples by the biopsy cannot accurately assess the diffuse MF. Furthermore, the endomyocardial biopsy is usually performed from the right ventricular side in the transplanted hearts and, thus, can not necessarily reflect LV information (1). Recently, the T1 time and ECV measured by CMR T1 mapping have emerged as a reliable non-invasive method for quantifying diffuse MF. However, previous studies showed high variability in native T1 time for assessing MF (20, 21). However, compared to native T1 time, myocardial ECV, which was derived from myocardial and blood pre- and post-contrast T1 relaxation time changes, has been validated as a preferred non-invasive method for quantifying actual diffuse MF in various cardiovascular diseases (10–13). Therefore, we selected the non-invasive CMR-ECV to measure diffuse MF in the patients after HT. This study proved that ECV was higher even in asymptomatic patients with HT with normal LVEF than in healthy controls, indicating the increased diffuse MF in the transplanted hearts. The increased MF in the transplanted hearts was consistent with a previous study that measured the MF through the histological biopsy or CMR T1 mapping (3–7, 22). AR, CAV, ischemic injure of the donor's heart, and postoperatively immunosuppressive therapy all may lead to MF of the transplanted hearts (2, 23, 24). Moreover, this study proved that CMR-ECV was positively correlated with time after HT, which corresponded to some other studies that indicated the MF may develop over time (3–5). Additionally, this study also proved that the ECV was correlated with the recipient age, which may suggest that recipient age would affect the allograft remodeling in the transplanted hearts. Furthermore, this study showed that the ECV did not correlate with ischemic time during transplantation, which was not in concordance with the study by Yuan et al. (22) but was in agreement with the study by Ide et al. (7). Therefore, the association between LVMF and the ischemic time needs to be further explored in the multicenter studies among broader research populations in the future.

### Correlation Between LV Myocardial Strain and CMR-ECV

The echocardiographic myocardial strain can accurately quantify ventricular mechanical function and it has emerged as a promising modality for predicting MF non-invasively and conveniently (14, 15). However, the correlation between the myocardial strain and MF in patients with post-HT has not been elucidated. Some studies have shown that increased LVMF measured by CMR-ECV was correlated with the decreased LV myocardial strain in a variety of heart diseases (16, 25–27). However, the studies about the association between 2D-myocardial strain and ECV show high variability and studies addressing which strain components might correlate best with ECV have not been seen. This is the first study to reveal the association between CMR-ECV and LV myocardial strain measured by 2D- and 3D-STE simultaneously in patients with HT.

This study showed that CMR-ECV both correlated with the 2D- and the 3D-LVGLS. A possible mechanism linking ECV to reduced systolic strain may be that the increased fibrosis leading to increased LV stiffness, which resulted in reduced end-diastolic muscle fiber length and, by the Frank–Starling law, reduced cardiac muscle contraction and systolic strain. The significant correlation between MF and GLS in the HT recipients in this study is in agreement with previous studies for other cardiovascular diseases (15, 16, 28). However, the increased ECV was not correlated with GCS or GRS in this study. This is possibly due to the fact that the LS mainly depends on the subendocardial layer and the subendocardial myocardium is sensitive to fibrosis (28). However, the CS mainly reflects the strain in the midmyocardium and RS is a change in the wall thickness of a length of the vertical line from a point on the endocardial border to a cross point on the epicardial border (29). In addition, patients with HT in our study had no severe complications at the time of CMR examination, such as AR and CAV in this study. Therefore, the extent of LV diffuse MF may be relatively mild in our subjects. Moreover, the early stage of MF mainly occurs in the endocardium and only LS can reveal this abnormality (29, 30). We believe that the aforementioned reasons may explain why only decreased GLS had a correlation with the increased ECV in the transplanted hearts.

Theoretically, compared with 2D-STE, 3D-STE is not affected by geometric modeling and out-of-plane motion, and it can evaluate the myocardial deformation in all the three spatial dimensions (17, 31). However, clinical testing and validation are needed to determine whether 3D-STE is superior to 2D-STE. The comparability of 2D- and 3D-STE has been studied extensively in healthy subjects and patients with various other cardiovascular diseases previously (17, 32, 33). However, the comparability of the association between 2D- and 3D-STE-derived strain and CMR evidence of diffuse MF has not been studied in patients post-HT simultaneously. This study made a direct comparison of strain values by 2D and 3D-STE in predicting the MF and revealed that 2D-LVGLS and 3D-LVGLS provides comparable results. The image quality and spatial resolution of 3D-STE are suboptimal than 2D-STE. The lower frame rate of 3D-STE may not be sufficient to accurately capture all the phases of a cardiac cycle. In addition, patients with HT had a higher heart rate. The aforementioned limitations of 3D-STE may lead to the conclusion that it is not superior to 2D-STE in correlation with CMR-ECV.

### Clinical Implications

This study proved that the level of diffuse MF defined by CMR-ECV was higher even in asymptomatic patients with HT than in healthy controls. Since MF is an adverse pathologic remodeling and can result in poor clinical outcomes, a reliable non-invasive method to monitor graft fibrosis would be clinically effective for the early identification of higher risk of patients with HT. This study showed that both 2D- and 3D-LVGLS were independently correlated with diffuse MF in HT recipients. Considering the correlation between 2D-STE or 3D-STE derived strain and MF is not robust and only presents up to 36% variations of MF. Therefore, CMR and cardiac biopsy cannot be replaced by these echocardiography modalities. Still this study suggested that measurements of 2D- and 3D-LVGLS may provide a valuable preliminary alternative non-invasive assessment of MF in the transplanted hearts, which may facilitate early risk stratification of HT recipients during follow-up examinations.

### Study Limitations

This study is a single-center study and is limited by a relatively small sample size. The HT recipients with AR and CAV at the time of CMR were not included in this study. So, the range of diffuse MF in this study was relatively narrow. In addition, there were no results of the histological biopsy in this study. (Nevertheless, ECV has been validated as a valuable surrogate marker for the assessment for the diffuse MF). Moreover, 3D-STE is highly independent of image quality and its frame rate was lower. Last, as the strain parameters are vendor dependent and not interchangeable, our results may not apply to other software algorithms.

## Conclusion

This study showed that CMR marker of diffuse MF was present in asymptomatic patients with HT and it was correlated with the 2D- and the 3D-LVGLS, not with LVGCS and LVGRS by echocardiography. The findings highlight the clinical superiority of the 2D- and the 3D-LVGLS over LVGCS, LVGRS, and other conventional parameters in predicting diffuse MF in HT recipients. 2D- and 3D-LVFLS may provide noninvasive tools for monitoring the development of adverse fibrotic remodeling during the serial follow-up of HT recipients.

## Data Availability Statement

The raw data supporting the conclusions of this article will be made available by the corresponding authors, without undue reservation.

## Ethics Statement

This study was approved by the Ethics Committee of Tongji Medical College, Huazhong University of Science and Technology. The patients/participants provided their written informed consent to participate in this study.

## Author Contributions

WS, BL, LZ, and MX: conception and design of study. WS, XS, JW, SZ, YZ, CW, YX, and YY: acquisition of data. WS, XS, JW, ND, GW, YL, and QL: analysis and/or interpretation of data. JW, BL, LZ, and MX: revised the manuscript. WS, XS, and JW: drafting the manuscript. QL, BL, LZ, and MX: revising the manuscript critically for important intellectual content. All authors contributed to the article and approved the submitted version.

## Funding

This study was supported by the National Natural Science Foundation of China (Grant Numbers: 81922033, 81671705, 81727805, and 81530056) and the Key Research and Development Program of Hubei (Grant Number: 2020DCD015).

## Conflict of Interest

The authors declare that the research was conducted in the absence of any commercial or financial relationships that could be construed as a potential conflict of interest.

## Publisher's Note

All claims expressed in this article are solely those of the authors and do not necessarily represent those of their affiliated organizations, or those of the publisher, the editors and the reviewers. Any product that may be evaluated in this article, or claim that may be made by its manufacturer, is not guaranteed or endorsed by the publisher.
